# Synthesis, Characterization, Crystal Structure, and Biological Studies of a Cadmium(II) Complex with a Tridentate Ligand 4′-Chloro-2,2′:6′,2′′-Terpyridine

**DOI:** 10.1155/2011/803292

**Published:** 2011-05-25

**Authors:** L. A. Saghatforoush, L. Valencia, F. Chalabian, Sh. Ghammamy

**Affiliations:** ^1^Chemistry Department, Payame Noor University, Tehran 19395-4697, Iran; ^2^Departamento de Quimica Inorganica, Facultad de Quimica, Universidade de Vigo, 36310 Vigo, Spain; ^3^Department of Biology, Islamic Azad University, Tehran North Campus, Tehran, Iran; ^4^Department of Chemistry, Imam Khomeini International University, Qazvin 34149-16818, Iran

## Abstract

A new Cd(II) complex with the ligand 4′-chloro-2,2′6′,2′′-terpyridine (Cltpy), [Cd(Cltpy)(I)_2_], has been synthesized and characterized by CHN elemental analysis, ^1^H-NMR, ^13^C-NMR, and IR spectroscopy and structurally analyzed by X-ray single-crystal diffraction. The single-crystal X-ray analyses show that the coordination number in complex is five with three terpyridine (Cltpy) N-donor atoms and two iodine atoms. The antibacterial activities of Cltpy and its Cd(II) complex are tested against different bacteria.

## 1. Introduction

**Figure 1:**
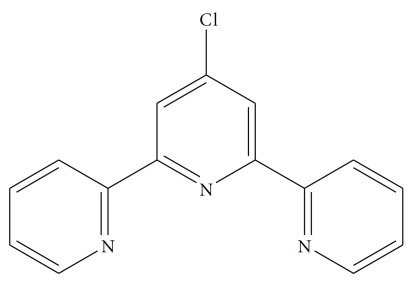
Structure of Cltpy ligand.

Terpyridine molecules with three nitrogen atoms acting as tridentate ligands to coordinate with various transition metal ions have been extensively studied [1, 2]. Coordination chemistry of the multitopic ligand of 2,2′:6′,2′′ terpyridine (tpy) particularly those substituted at the 4-position has been recently attracting growing attention in the design of the supramolecular building blocks based on the metaldirected self-assembly [3–26]. The 4′-chloro-2,2′:6′,2′′-terpyridine (Cltpy) ligand (Figure 1) contains one widely used tpy coordinative site and the other Cl site at the 4′-position. These two sites are able to bind with different metal ions, thus leading to the coordination polymers with various frameworks [21]. Tpy can bind to both low- and high-oxidation state metal ions, almost always in tridentate fashion [27–32]. The synthesis of tpy derivatives has been extensively studied by Constable's group, and varieties of substituted tpy compounds have been reported [19, 20]. For example, they offered interesting prospects for metal-activated drug delivery system, where the activity could be switched by metal-ion coordination through the study of the interactions between bioreceptors and ligand with sugar substituents [19, 20]. In clinical applications and biochemistry, functionalized terpyridines have found a wide range of potential uses, ranging from colorimetric metal determination to DNA binding agents [33]. Metal terpyridine complexes due to the binding to nucleic acids have the ability or potential to serve as anticancer, antibacterial, and antiparasitic drugs [34–39]. The exact mechanisms are not known in some cases and may involve protein binding or membrane binding. The interaction Cd(II) ion with biomolecules is one of the most studying fields in coordination chemistry, and cadmium is a very toxic metal and widely used in many industrial processes [40, 41]. In this research we used new tridentate ligand 4′-chloro-2,2′:6′,2′′-terpyridine (Cltpy) that has been used for synthesis of a new cadmium(II) complex. The structural and biological properties of this new complex have been studied.

## 2. Experimental

### 2.1. Materials and Measurements

All chemicals were reagent grade and used without further purification. Elemental analyses (CHN) were performed using a Carlo ERBA model EA 1108 analyzer. FT-IR spectra were collected on a Shimadzu prestige 21 spectrophotometer in the range of 4000–400 cm^−1^ using KBr pellets. ^1^H and ^13^C NMR spectra were recorded with a Bruker spectrometer at 250 MHz in D_6_-DMSO.

### 2.2. Preparation of [Cd(Cltpy)(I)_2_]

4′-chloro-2,2′:6′,2′′-terpyridine (0.268 g, 1 mmol) was placed in one arm of a branched tube, cadmium(II) acetat (0.264 g, 1 mmol) and potassium iodide (0.332 g, 2 mmol) in the other. Methanol was carefully added to fill both arms, the tube was then sealed, and the ligand-containing arm was immersed in a bath at 60°C, while the other remained at ambient temperature. After two days, the light brown crystals that had deposited in the cooler arm were filtered off, then washed with diethylether, and air dried. Yield: 71%. Analysis: found: C: 28.38, H: 1.51, N: 6.59%. Calculated for C_15_H_10_CdClI_2_N_3_: C: 28.42, H: 1.59, N, 6.63%. **IR** (cm^−1^) selected bonds: 679(w), 798(s), 822(m), 1005(m), 1142(w), 1405(s), 1475(m), 1545(s), 1582(s), 3042(w), 3065(w). **^1^H NMR** (DMSO, *δ*): 7.49 (t, 2H), 8.011 (t, 2H), 8.404 (s, 2H), 8.684 (m, 4H) ppm. **^13^C NMR** (DMSO, *δ*): 120.46, 121.06, 124.34, 135.31, 140.65, 146.52, 152.12, 155.36 ppm.

### 2.3. Antibacterial Activity Test

In vitro activity test was carried out using the growth inhibitory zone (well method) [42–45]. The potency of components was determined against the three Gram-positive bacteria, *Streptococcus pyogenes* (RITCC 1940), *Staphylococcus aureus* (RITCC 1885), and *Bacillus anthracis *(RITCC 1036), and also against the three Gram-negative bacteria, *Klebsiella pneumonia* (RITCC 1249),* Escherichia coli* (RITCC 1330), and *Pseudomonas aeruginosa *(RITCC 1547). Microorganisms (obtained from enrichment culture of the microorganisms in 1 mL Muller-Hinton broth incubated at 37°C for 12 h) were cultured on Muller-Hinton agar medium. The inhibitory activity was compared with that of standard antibiotics, such as gentamicin (10 *μ*g). After drilling wells on the medium using a 6 mm cork borer, 100 *μ*L of solution from different compounds were poured into each well. The plates were incubated at 37°C overnight. The diameter of the inhibition zone was measured as precisely as possible. Each test was carried out in triplicate, and the average was calculated for inhibition zone diameters. A blank containing only DMSO showed no inhibition in a preliminary test. The macrodilution broth susceptibility assay was used for the evaluation of minimal inhibitory concentration (MIC). The use of 12 test tubes is required by the macrodilution method. By including 1 mL Muller-Hinton broth in each test and then adding 1 mL extract with concentration 100 mg/mL in the first tube, we made a serial dilution of this extract from the first tube to the last tube. Bacterial suspensions were prepared to match the turbidity of 0.5 McFarland turbidity standards. Matching this turbidity provided bacterial inoculums concentration of 1.5 × 10^8^ cfu/mL. Then 1 mL of bacterial suspension was added to each test tube. After incubation at 37°C for 24 h, the last tube was determined as the minimal inhibitory concentration (MIC) without turbidity.

### 2.4. X-Ray Crystallography

#### 2.4.1. Structure Determination

Data collection for X-ray crystal structure determination was performed on a STOE IPDS I/II diffractometer using graphite-monochromated Mo-K_*α*_ radiation ($\lambda =0.71073\;\overset{´}{Å}$). The data were corrected for Lorentz and polarization effects. A numerical absorption correction based on crystal-shape optimization was applied for all data. The programs used in this work are Stoe's X-Area, including X-RED and X-Shape for data reduction and absorption correction, and the WinGX suite of programs, including Sir-92 and Shelxl-97 for structure solution and refinement [46]. The hydrogen atoms were placed in idealized positions and constrained to ride on their parent atom. The last cycles of refinement included atomic positions for all atoms, anisotropic thermal parameters for all nonhydrogen atoms, and isotropic thermal parameters for all hydrogen atoms. Materials for publication were prepared by using Mercury and Ortep-3 [47, 48]. The summary of the crystal data, experimental details, and refinement results of complex is listed in Table 1.

## 3. Results and Discussion

### 3.1. Spectroscopic Studies

The reaction of CdX_2_ (X: nitrate and acetate) with 4′-chloro-2,2′:6′,2′′-terpyridine (Cltpy) and potassium iodide yielded crystalline material formulated as [Cd(Cltpy)(I)_2_]. The IR spectra display characteristic absorption bands for the tpyCl ligands. The relatively weak absorption bands at around 3042–3065 cm^−1^ are due to the C–H modes involving the aromatic ring hydrogen atoms. The absorption bands with variable intensity in the frequency range 1400–1620 cm^−1^ correspond to aromatic ring vibrations of the tpyCl ligand [29–40, 49]. 

The ^1^H-NMR spectra of DMSO solutions of complex at room temperature show two triplets, a singlet, and a multiplet for the aromatic protons of Cltpy ligand. The ^13^C-NMR spectra of DMSO solutions of these compounds show eight distinct bands assigned to the aromatic carbon atoms of the pyridine rings of the Cltpy ligand.

### 3.2. Structural Analysis

**Figure 2:**
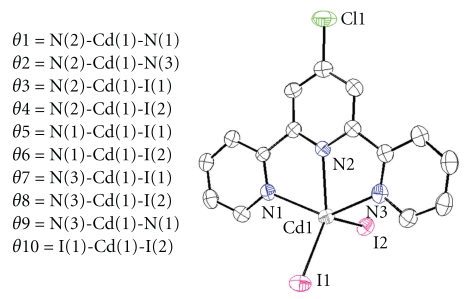
Molecular structure of [Cd(Cltpy)I_2_] including the atom numbering scheme. All hydrogen atoms have been omitted for clarity.

The solid-state structure of compound was determined by single-crystal X-ray diffraction. Crystal and structure refinement data of the compound are given in Table 1. X-ray crystal analysis reveals that the compound crystallizes in monoclinic with space group P2(1)/c. The crystal structure of compound consists of monomeric units. Each cadmium atom chelated by three Cltpy nitrogen atoms and two iodine atoms (Figure 2). The resulting coordination number of five is augmented with CdN_3_I_2_ molecule core. Selected bond lengths and angels of complex are given in Table 2.

 The complex structure was described in detail. Five coordinate complexes with chelating ligands can exhibit either square pyramidal or trigonal bipyramidal geometries, and the particular case is influenced by both steric and electronic factors. The variation of five coordinate species between square pyramidal and trigonal bipyramidal is quantified using *τ* values calculated by (1), where *β* is the largest X–Cu–X bond angle and *α* is the second largest X–Cu–X angle. For the regular square pyramidal structures the trigonality parameter *τ* will be zero, and it increases to 1.0 as the trigonal bipyramidal distortion increases [50–52]:

(1) $$\tau \;=\;\frac{\left(\beta -\alpha \right)}{60}.$$

The new [Cd(Cltpy)(I)_2_] complex reported herein takes on a slightly distorted square pyramidal structure as evidenced by the *τ* value of 0.029. Definition of the bond angles (*θ*) in [Cd(Cltpy)(I)_2_] complex is illustrated in Figure 1. The Cd1–N1, Cd1–N2, and Cd1–N3 bond lengths are within the normal range of Cd–N bonds. The average bond length of Cd–I is 2.7417(7) Å that is slightly shorter than the reported bond length [53]. The I2 occupies the apical position in complex at relatively longer distance. The rigid character of the tpy bite leads Cd–N bond lengths in the complex to follow the general trend of Cdtpy complexes, that is, to have their Cd–N central bond slightly shorter than the Cd–N lateral [54, 55]. The tpy ligand is not planar; due to coordination strain the molecule is bent into a concave shape, the central pyridyl group subtending dihedral angles of 5.85(1) and 9.19(1)° to the lateral ones.

**Figure 3:**
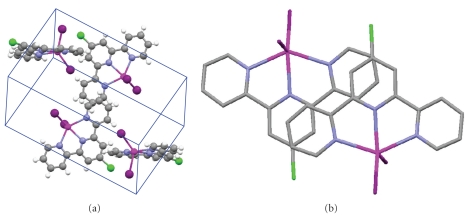
(a) The unit cell of [Cd(Cltpy)I_2_]. (b) Close-up view of the aromatic *π*-*π* contacts showing ligand overlap (slipped face-to-face interaction).

 This complex has some intermolecular interactions. Intermolecular interactions in crystal of [Cd(Cltpy)I_2_] complex are shown in Table 3. There are aromatic *π*-*π* stacking interactions between the parallel aromatic rings of the 4′-chloro-2,2′:6′,2′′-terpyridine (Cltpy) ligands as seen in Figure 3(b). 

 The unit cell of complex is shown in Figure 2. Molecules occupied half of tetrahedral holes and used zinc blend system that is not a closed packed system.

### 3.3. Antibacterial Activity

The antibacterial activities of Cltpy and its Cd(II) complex are shown in Table 4. The free ligand has considerable activity against* Staphylococcus pyogenes*, *Bacillus anthracis,* and *Pseudomonas aeruginosa* (inhibitory zones ≥20 mm) but has moderate activity against *Escherichia coli* and* Streptococcus aureus *(inhibitory zones ≤15 mm) [42, 43]. In comparison with free Cltpy ligand, the complex has more activity against* Escherichia coli* (more inhibitory zones), but it is less active against* Staphylococcus pyogenes*, *Bacillus anthracis,* and *Streptococcus aureus*, [45]. It is should be noticed that the antibacterial activity of Cltpy ligand is higher than standard antibiotic (gentamicin) against* Pseudomonas aeruginosa*,* Streptococcus pyogenes,* and* Klebsiella pneumonia*. 

Against* Escherichia coli*, antibacterial activity of complex is higher than Cltpy ligand. The higher activity of complex may be explained on the basis of chelation theory [42, 43]. Also the better antibacterial activity of complex is probably due to the existence of I^−^anion in its structure [44, 45]. The quantitative assays gave MIC values in the range 50–100 mg ml^−1^ (Table 4) that confirmed the previous results.

## 4. Conclusion

New multidentate ligand, 4′-chloro-2,2′:6′,2′′-terpyridine (Cltpy), has been used for preparation of an inorganic complex. A new Cd(II) complex [Cd(Cltpy)I_2_] has been synthesized and characterized by CHN elemental analysis, ^1^H-NMR, ^13^C-NMR, and IR spectroscopy, and structurally analyzed by X-ray single-crystal diffraction. The single-crystal X-ray analyses show that the coordination number in this complex is five with three terpyridine (Cltpy) N-donor atoms and two iodine atoms. The antibacterial activities of Cltpy and its Cd(II) complex are tested against different bacteria. The complex has good activity against all tested bacteria. Against* Escherichia coli*, antibacterial activity of complex is higher than Cltpy ligand. The higher activity of complex may be explained on the basis of chelation theory.

##  Additional Data

CCDC reference number 799987 contains the additional crystallographic data for this paper. These data can be obtained free of charge at http://www.ccdc.cam.ac.uk/conts/retrieving.html.

## Figures and Tables

**Table 1:** Crystallographic data of [Cd(Cltpy)I_2_ complex.

Identification code	Cd (terpy-Cl) I_2_ (7)
Empirical formula	C15 H10 Cd Cl I2 N3
Formula weight	633.91
Colour	light brown
Temperature	293(2) K
Wavelength	0.71073 A
Crystal system	Monoclinic
Space group	P2(1)/c
Unit cell dimensions	*a* = 11.4865(10) Å
	*b* = 8.9326(7) Å
	*c* = 17.7570(14) Å
	*α* = 90°
	*β* = 94.242(2)°
	*γ* = 90°
Volume	1817.0(3) Å^3^
Z	4
Density (calculated)	2.317 g cm^−3^
Absorption coefficient	4.745 mm^−1^
*F*(000)	1168
Crystal size	0.27 × 0.14 × 0.13 mm^3^
Theta range for data collection	1.78 to 25.02°
Index ranges	−11 ≤ *h* ≤ 13
	−10 ≤ *k* ≤ 10
	−21 ≤ *l* ≤ 19
Reflections collected	9236
Independent reflections	3184
Absorption correction	Empirical
Max. and min. transmission	0.5774 and 0.3606
Refinement method	Full-matrix
	least-squares on *F* ^2^
Data/restraints/parameters	3184/0/199
Goodness-of-fit on *F* ^2^	1.063
Final *R* indices [*I* > 2**σ**(*I*)]	*R*_1_ = 0.0309
	*wR*_2_ = 0.0747
*R* indices (all data)	*R*_1_ = 0.0504
	*wR*_2_ = 0.0862
Largest diff. peak, hole	0.802 and −0.708e· Å^−3^

**Table 2:** Selected bond lengths/Å and angles/°.

[Cd(Cltpy)I_2_]			
Cd1–N1	2.373(5)	*θ*3	135.19(11)
Cd1–N2	2.331(5)	*θ*4	106.17(11)
Cd1–N3	2.381(5)	*θ*5	101.35(12)
Cd1–I1	2.7311(8)	*θ*6	101.13(13)
Cd1–I2	2.7523(7)	*θ*7	101.33(13)
		*θ*8	99.34(13)
*θ*1	69.24(17)	*θ*9	130.94(18)
*θ*2	68.81(18)	*θ*10	118.63(2)
			

**Table 3:** Intermolecular interactions in crystals of [Cd(Cltpy)I_2_] complex.

A*⋯*H-B	H*⋯*A/Å	B*⋯*A/Å	B-H*⋯*A/°
			
I2*⋯*H14–C14(−*x*, *y*, − *z* + 1/2)	2.937	3.620(2)	166.16
I2*⋯*H12–C12(−*x*, *y*, − *z* + 1/2)	3.130	3.435(2)	161.15
I2*⋯*H13–C13(−*x*, *y*, − *z* + 1/2)	3.163	3.308(2)	135.50
C2(N1C1–C5)*⋯*C8(N2C6–C10)		3.472(3)	
Centroid*⋯*centroid (N1C1–C5)*···*(N2C6–C10)		3.818(2)
Centroid*⋯*centroid (N3C11–C15)*···*(N3C11–C15)		4.275(2)

**Table 4:** Antibacterial activities (zone of growth inhibition and minimal inhibitory concentrations) of Cltpy ligand and Cd (II) complex and gentamicin (as a standard compound).

Method	Main compounds	Microorganisms					
		*Klebsiella pneumonia *(−)	*Escherichia coli *(−)	*Pseudomonas aeruginosa *(−)	*Streptococcus pyogenes *(+)	*B. anthracis *(+)	*Staphyeococcus aureus *(+)
Growth Inhibitory zone [mm]	Cltpy	10	15	20	30	25	15
	Complex	10	20	20	10	12	10
Standard	Gentamicin	20	25	15	13	32	20
							
Minimum inhibitory concentration (mg/mL) (MIC)	L	100	100	50	6.25	12.5	100
	Complex	100	50	50	100	100	100
							
